# Haemophilia and access to gene therapy

**DOI:** 10.1016/j.ebiom.2025.105712

**Published:** 2025-04-15

**Authors:** Johnny Mahlangu

**Affiliations:** Department of Molecular Medicine and Haematology, Faculty of Health Sciences, University of the Witwatersrand, Johannesburg, South Africa

Mutations in the clotting factor genes (*Factor 8 or 9 genes*) lead to impaired thrombin generation, resulting in haemophilia, characterised by haemarthroses. Over the past two decades, the evolving landscape of haemophilia therapies has been remarkable, with replacement treatments, non-factor therapies, and, more recently, gene therapy products enriching the therapeutic options available for managing haemophilia.^1^

The FDA and EMEA have approved several haemophilia adeno-associated virus (AAV)-delivered gene therapies, which provide various advantages such as a functional cure, enhanced quality of life, and a reduced treatment burden. However, real-world experience with haemophilia gene therapy (HGT) products is limited, and access in resource-constrained health settings encounters several barriers, including product-related and health-system constraint factors.

Important product-related barriers to HGT include the high prevalence of pre-existing anti-AAV antibodies, the steep cost of products, and the uncertain long-term durability of expression. In resource-constrained settings, HGT barriers may encompass inaccessible products, a lack of gene therapy infrastructure and expertise, poorly defined regulatory and policy frameworks, as well as cultural and socioeconomic hurdles, along with ethical and privacy concerns.^2^ To benefit individuals with haemophilia, both product and health system obstacles to HGT must be tackled.

The delivery of the haemophilia *F8 gene* using lentivirus has advanced beyond proof of concept, as shown by recent phase 1 results in patients with haemophilia A.^3^ These findings indicated that lentivirus-transduced autologous haemopoietic stem cells led to stable FVIII expression. The lentivirus delivery method removes the necessity to screen for anti-AAV antibodies and does not involve using steroids to manage unpredictable transaminitis, addressing both limitations of AAV-mediated gene therapy.

To address the unfulfilled long-term durability requirement, the BE-Bio B-cell medicine programme combines long-lived allogeneic plasma cells with a CRISPR/Cas guide RNA, which inserts into the F9 transgene at the CCR5 locus *in vivo*.^4^ The gene-edited B-cells demonstrated efficient secretion of FIX in preclinical studies. This approach offers several advantages over AAV, including a sustainable and consistent level of expression, eliminating the need for preconditioning, allowing for redosing and titration, and being accessible at all ages, including paediatrics.

Substantial infrastructure and expertise were established during the COVID-19 pandemic, and these are now being repurposed to address unmet needs in both genetic and non-genetic diseases. When applied to HGT, this customised approach will lower manufacturing costs and enhance its affordability and accessibility.^5^ Over time, HGT may become as accessible as CAR-T gene therapies currently available worldwide, including in resource-limited settings. Although the initial generation of HGT began with some pessimism, evolving data suggest a more optimistic future.

## Contributors

I am the sole author.

## Declaration of interests

JM has received research grant/research support from Biomarin, CSL Berhing, Novo Nordisk, Pfizer, Roche, Sanofi, Spark and Vega Therapeutics has been a consultant/scientific board for Biomarin, CSL Behring, Novo Nordisk, Roche, Takeda, Sanofi, Spark and Vega Thrapeutics.
